# Mechanical Injury among Medicolegal Cases in the Department of Emergency in a Tertiary Care Centre: A Descriptive Cross-sectional Study

**DOI:** 10.31729/jnma.7914

**Published:** 2022-12-31

**Authors:** Sidarth Timsinha, Sudhir Raman Parajuli

**Affiliations:** 1 Department of Forensic Medicine, Birat Medical College Teaching Hospital, Biratnagar, Morang, Nepal; 2 Department of Forensic Medicine, Manipal College of Medical Sciences, Fulbari, Pokhara, Nepal

**Keywords:** *accidents*, *documentation*, *forensic medicine*, *injuries*, *Nepal*

## Abstract

**Introduction:**

A medico-legal issue arises whenever an injured person visits a hospital. Therefore, all physicians who treat such patients have a legal duty to accurately document injuries as part of medical treatment. The study aimed to find out the prevalence of mechanical injury among medicolegal cases in the Department of Emergency in a tertiary care centre.

**Methods:**

A descriptive cross-sectional study was conducted among patients admitted to the Emergency Department of a tertiary centre from May 2018 to April 2020 after obtaining ethical approval from the Institutional Review Committee (Reference number: 356). Convenience sampling method was used among patients who met the eligibility criteria. All relevant data were extracted using hospital records. Point estimate and 95% Confidence Interval were calculated.

**Results:**

Out of 3486 medicolegal cases registered in the Department of Emergency, 856 (24.55%) (28.37-29.96, 95% Confidence interval) were cases of mechanical injuries. Males 616 (71.96%) outnumbered females for all types of mechanical injuries. Road Traffic Accident 527 (61.56%) was the leading cause of injury and laceration 351 (29.52%) was the most common pattern of injury. The majority 628 (73.36%) of the injuries were simple in nature.

**Conclusions:**

The prevalence of mechanical injuries among medicolegal cases was similar in comparison to other studies done in similar settings. The majority of the injuries were caused by traffic accidents, which could have been prevented if a safe system approach to road safety was followed.

## INTRODUCTION

The Criminal Code of Nepal 2074 defines injury as any harm caused by an act of commission or omission resulting in damage to an individual's body, mind, dignity, right, or a breach of contract.^1^ Mechanical injury is any bodily harm caused by a breach in the natural continuity of any body tissue caused by the application of mechanical force. The mechanical force can be blunt force, sharp force, or firearms.^2^

Injury and violence cause five million deaths annually in the world which is around 9% of global mortality.^3^ Injuries are generally the predictable outcome of specific circumstances and are thus largely preventable.^4^ Despite being predictable and largely preventable there are only a few studies on trauma patients in Nepal who visit hospitals for treatment.

The study aimed to find out the prevalence of mechanical injury among medicolegal cases admitted to the emergency department in a tertiary care centre.

## METHODS

A descriptive cross-sectional study was conducted among patients of medicolegal cases in the Department of Emergency in a tertiary centre from May 2018 to April 2020 after obtaining ethical approval from the Institutional Review Committee (Reference number: 356). All the patients admitted to the emergency department with medicolegal cases were included in the study. Patients with missing medical record files, cases with incomplete clinical information and brought dead cases were excluded from the study. Convenience sampling was done. The sample size was calculated using the following formula:


$$\begin{array}{ll} \text{n} & ={\text{Z}}^{2}\times \frac{\text{p}\times \text{q}}{{\text{e}}^{\text{2}}}\\ & =1.96^{2}\times \frac{0.50\times 0.50}{0.035^{2}}\\ & =784 \end{array}$$

Where,

n = minimum required sample size
Z= 1.96 at 95% Confidence Interval (CI)
p = prevalence taken as 50% for maximum sample size calculation
q = 1-p
e = margin of error, 3.5%

The minimum sample size calculated was 784. However, a sample size of 856 was taken for the study. Data were extracted from the medicolegal register maintained in the emergency department, hospital admission records, case notes, and statements of the relatives recorded. Data regarding sociodemographic factors, type of injury, the severity of the injury, causative weapon or force used, site of injury, manner of injury, and the cause of injury and outcome were recorded in a Performa during data collection.

Data were entered in Microsoft Excel and analyzed in IBM SPSS Statistics version 21.0. Point estimate and 95% CI were calculated.

## RESULTS

Among 3486 medicolegal cases admitted to the emergency department during the study period, the prevalence of mechanical injury was 856 (24.56%) (23.13-25.99, 95% CI). The mean age of the patients was 29.17±11.82 years. Among 856 cases, 616 (71.96%) were male and 240 (28.04%) were female with a male-to-female ratio of 3:1. The majority of injuries 442 (51.64%) were seen in the 16-30 years age group followed by age group 31-45 years 211 (24.65%) (Table 1).

**Table 1:** Socio-demographic findings (n= 856).

Age group (years)	n (%)
0-15	64 (7.48)
16-30	442 (51.64)
31-45	211 (24.65)
46-60	104 (12.15)
>60	35 (4.09)

Lacerations 351 (41%), were the commonest pattern of injury followed by contusions 259 (30.26%), and incised wounds 196 (16.48%) (Table 2).

**Table 2:** Pattern of mechanical injuries (n= 856).

Pattern	No of cases n (%)
Abrasion	179 (20.91)
Contusion	259 (30.26)
Laceration	351 (41.00)
Incised wound	196 (22.90)
Chop wound	8 (0.93)
Gunshot wound	-
Stab wound	5 (0.58)
Fractures	144 (16.82)
Intra cranial injuries	44 (5.14)
Others	3 (0.35)

* Number exceeds the number of cases due to multiple injuries

Overall, Road Traffic Accidents (RTAs) 527 (61.57%) was the leading cause of mechanical injury, followed by physical assault 186 (21.72%) and fall injuries 139 (16.23%). RTA 283 (33.06%) and physical assault 142 (16.59%) were common in the 16-30 years age group, while fall injuries 61 (7.13%) were common in the 4060 years age group (Table 3).

**Table 3:** Distribution of cause of injury according to age group (n= 856).

Age group (years)	Causes of injury					
	RTA (n= 527)	Physical assault (n= 186)	Fall (n= 139)	Occupational (n= 1)	Sports injury (n=3)
	n (%)	n (%)	n (%)	n (%)	n (%)
0-15	49 (5.72)	-	13 (1.52)	-	2 (0.23)
16-30	283 (33.06)	142 (16.59)	16 (1.87)	-	1 (0.12)
31-45	123 (14.37)	42 (4.91)	45 (5.26)	1 (0.12)	-
46-60	41 (4.79)	2 (0.23)	61 (7.13)	-	-
>60	31		4		
	(3.62)		(0.47)		

Overall extremities 534 (44.91%) was the commonest site of injury followed by head and neck 356 (29.94%). Medicolegally, all injuries were classified as simple, grievous, life-threatening, or grievous and life-threatening as shown in cross-tabulation (Table 4).

**Table 4:** Distribution of severity of the injury (n= 856).

Severity	n (%)
Simple	656 (76.64)
Grievous	176 (20.56)
Grievous and life-threatening	8 (0.93)
Life-threatening	16 (1.87)

In our study death occurred in 69 (8.06%) of the cases with a high fatality rate of 65 (12.33%) in RTA cases.

## DISCUSSION

During the study period, 856 cases of mechanical injuries were reported to the Emergency Department of Manipal Teaching Hospital (MTH) Pokhara giving a prevalence of 24.55%. Overall, the majority of the injury patients were young males, in the age group of 16-30 years 51.64% which is similar to a study.^5^ One of the reasons for this could be young males being more active and busy outdoors and often involved in risk-taking activities. The younger age groups of 1630 years 51.64% were more vulnerable to RTAs and physical assault, whereas the older age group of 4660 years 43.88% was more susceptible to fall injuries which is similar to the findings of a study.^6^ The variety and range of activities in this age group increases the risk of injury and as a productive segment of society, this indicates a huge economic loss. This confirms that while age is a major risk factor for many injuries, its influence varies among specific gender and age group. In our study lacerations of 30% were seen as the commonest pattern of injury, followed by contusions of 21.78% and incised wounds of 16.48%. In different communities, the pattern of injury varies depending on the cause, socioeconomic status, and other demographic factors. In a study contusion, laceration, and abrasion was the most common pattern of injury.^6^ In a similar study bruises and superficial cuts 56.7% were found to be the common pattern of injury.^7^

RTA was the leading cause of injury accounting for 61.56% of total cases, similar to a finding in a study.^6^ This could be due to factors like rapid growth in the number of automobiles, poor quality roads, and ineffective enforcement of traffic safety regulations. Also, distracted driving, such as using mobile phones while driving and driving under the influence of alcohol or drugs, may contribute significantly to the risk of transportation injuries. This can be minimized by incorporating injury prevention measures into children's school curricula, as well as RTA prevention guidelines, particularly targeting boys during their formative years. However, in a study conducted in one of Nepal's seven federal provinces, RTA was the second most common cause of injury, and the leading cause of injury there was fall injury.^8^ This disparity could be because their study area is one of the most remote and inaccessible areas in Nepal, reachable only by foot or small aircraft. Due to fewer motor vehicles and limited road access, the incidence of RTA could be less. However, RTA continues to be the leading cause of death and disability in the lowlands of Nepal.^9^

In RTA cases, more than half 63.20% of the injuries were caused due to falls on the road. In cases of physical assault, blunt weapons 38.11% used were in the form of fists, kicks, and stones, followed by iron rods and wooden sticks which is similar to a study conducted in Nepal.^10^ In contrast another study in Nepal reported wooden sticks and clubs 21.5% as commonly used weapons in physical assault, followed by kicks and punches (20.6%).^11^

The commonest site of injury was extremities at 45% a finding similar to a study.^12^ However, this finding contrasted with head injuries, seen in other parts of the world.^13^ The use of extremities may have come into play during the time of defence or at the time of the vehicular accident or during a fall to protect the vital parts of the body.

Medicolegally, all injuries were classified based on their severity as simple, grievous, life-threatening, and grievous life-threatening. Grievous injuries are described in the National Penal Code Act 2017 of Nepal.^14^ However, life-threatening injuries are not well detailed in the National Penal Code Act 2017 of Nepal, but as previously described in the literature are those injuries with eminent risk of death if treatment is not received promptly that includes cases like traumatic brain injuries, skull fractures, and so on.^15^

The limitations of this study was a cross-sectional study from a single centre so the results cannot be extrapolated to a national level. Future large-scale multi-centre studies with a large sample size are recommended.

## CONCLUSIONS

Our study found the prevalence of mechanical injuries similar to other studies done in similar settings. The leading cause of injury was road traffic accidents with a high fatality rate. The majority of these injuries could have been prevented with the use of proactive measures, including post-crash response training, safer roads, vehicles, and users, as well as education about road safety management. In addition to implementing proper preventive measures, the injury-treating doctor should always examine the wound carefully. This can be beneficial not only in evaluating and interpreting such injuries but also in assisting law enforcement agencies, particularly the judiciary, in reaching logical decisions.
